# Supplementary material for: DMRforPairs: identifying Differentially Methylated Regions between unique samples using array based methylation profiles
Source: BMC Bioinformatics. 2014 May 15;15:141. doi: 10.1186/1471-2105-15-141 (PMC4046028; doi:10.1186/1471-2105-15-141)
Supplement: Additional file 2 — DMRforPairs output for the comparison of A431-MCF7 and NA17018-NA17105. Please start from the HTML files in each folder. Available via the BMC Bioinformatics website. [file 1471-2105-15-141-S2.zip › 1394847754114233_MOESM2_ESM/A431_MCF7/figures/36661.pdf]

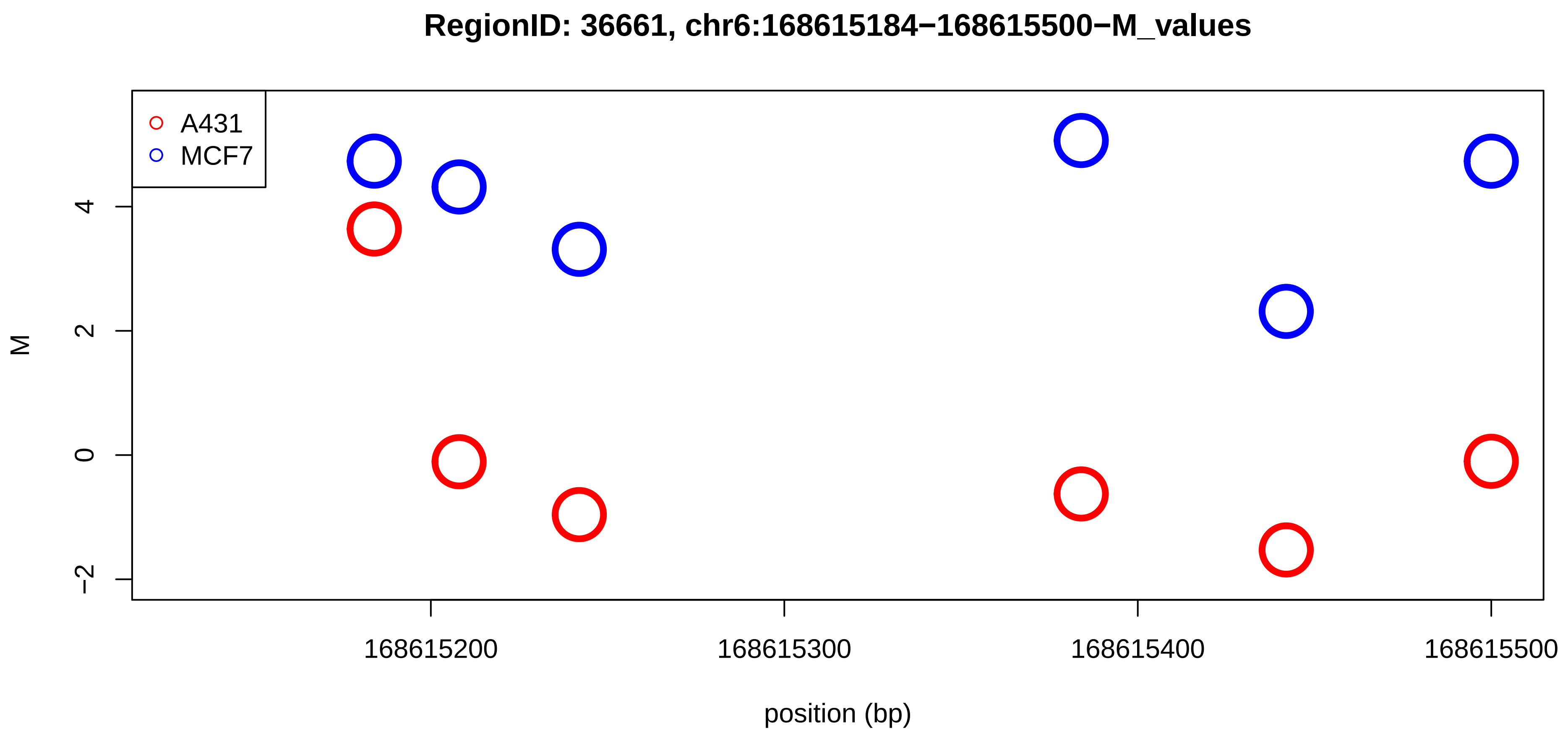
RegionID: 36661, chr6:168615184−168615500−M_values
A431
MCF7
M
position (bp)
168615200
168615300
168615400
168615500
−2
0
2
4

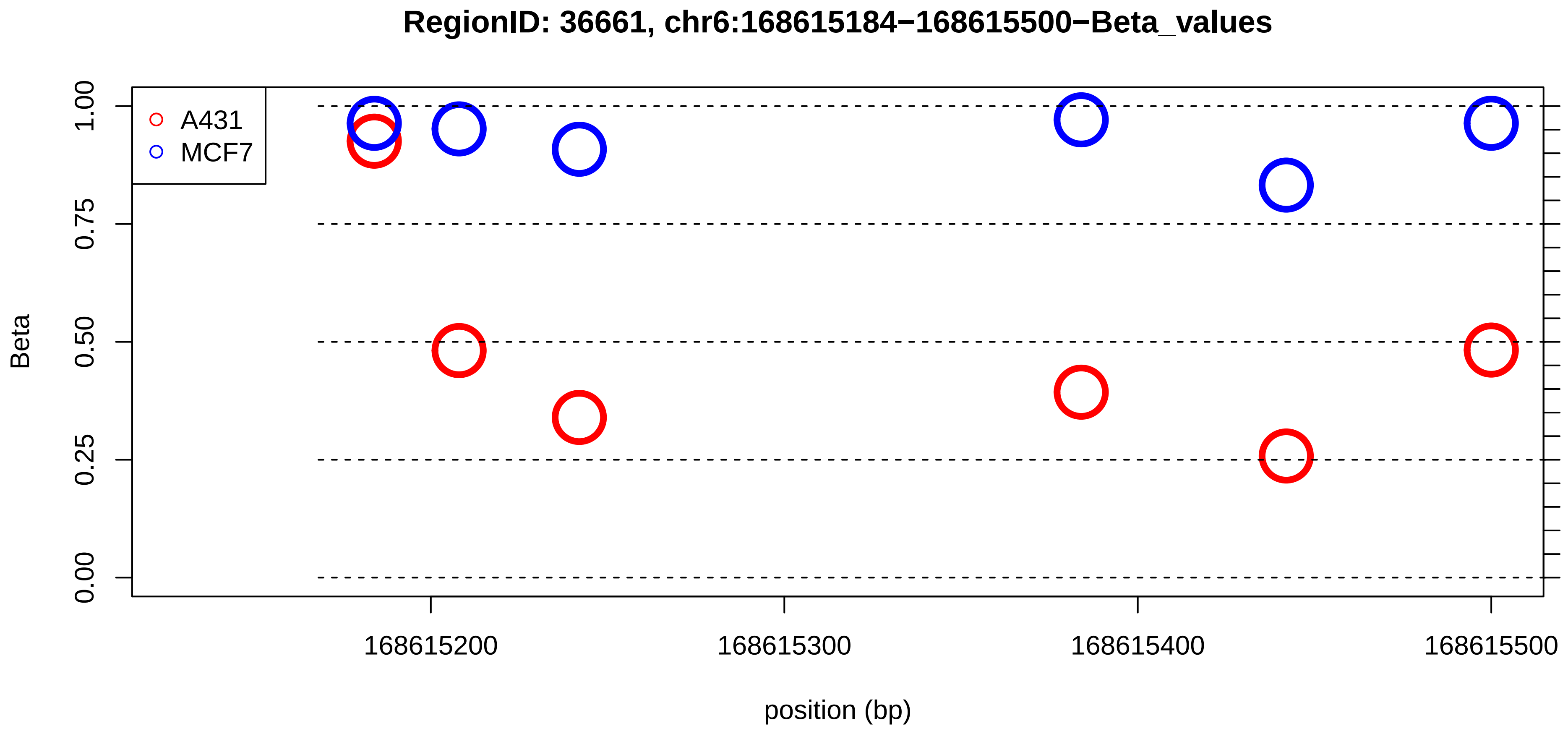
RegionID: 36661, chr6:168615184−168615500−Beta_values
A431
MCF7
Beta
position (bp)
168615200
168615300
168615400
168615500
0.00
0.25
0.50
0.75
1.00

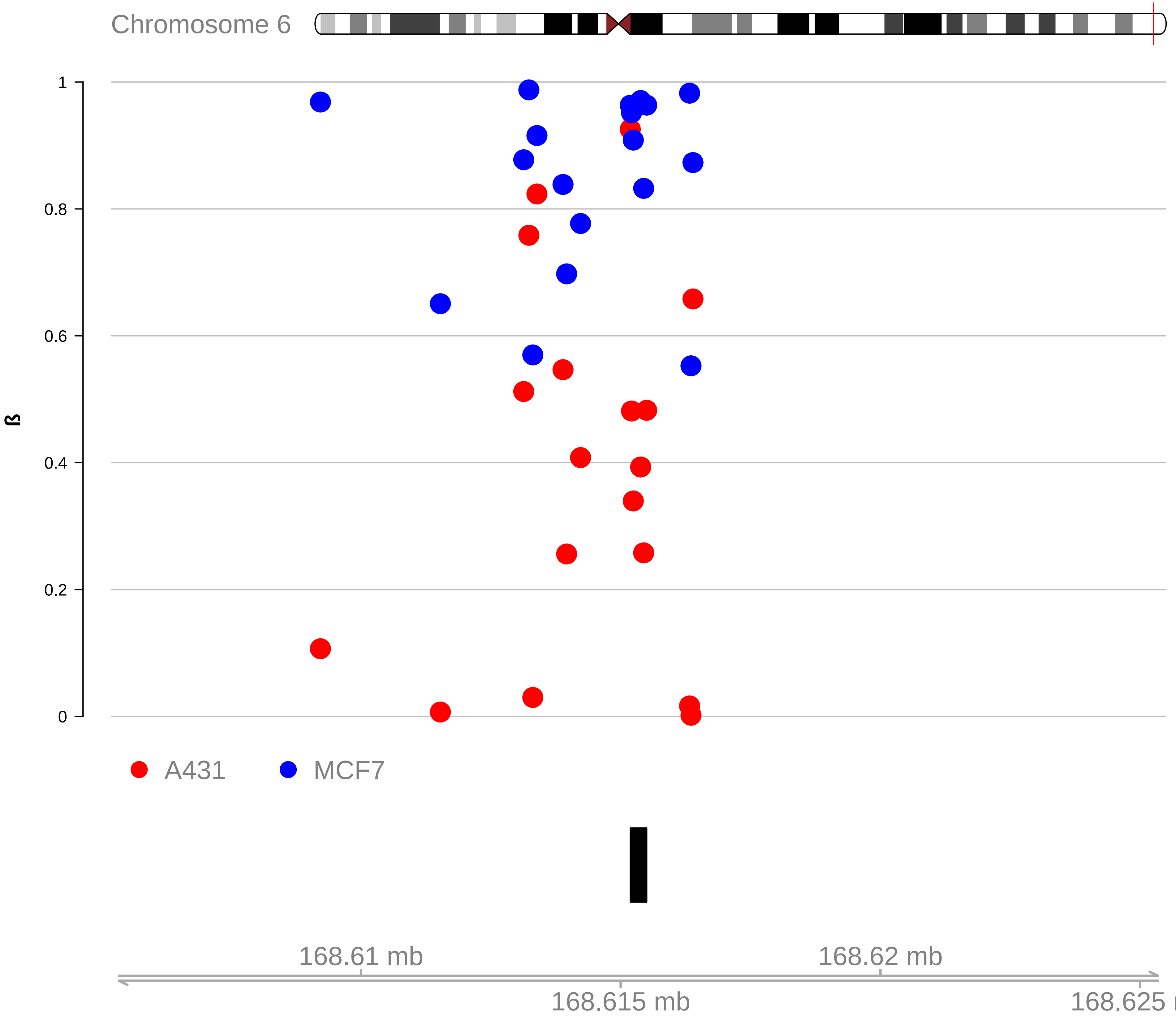

Chromosome 6
1
0.8
0.6
0.4
0.2
0
ß
A431
MCF7
168.61 mb
168.62 mb
168.615 mb
168.625 m
